# Flux focusing with a superconducting nanoneedle for scanning SQUID susceptometry

**DOI:** 10.1038/s41378-023-00553-9

**Published:** 2023-06-12

**Authors:** B. K. Xiang, S. Y. Wang, Y. F. Wang, J. J. Zhu, H. T. Xu, Y. H. Wang

**Affiliations:** 1 State Key Laboratory of Surface Physics and Department of Physics, Fudan University, 200433 Shanghai, China; 2 Shanghai Research Center for Quantum Sciences, 201315 Shanghai, China

**Keywords:** Nanosensors, Electronic properties and materials

## Abstract

A nanofabricated superconducting quantum interference device (nano-SQUID) is a direct and sensitive flux probe used for magnetic imaging of quantum materials and mesoscopic devices. Due to the functionalities of superconductive integrated circuits, nano-SQUIDs fabricated on chips are particularly versatile, but their spatial resolution has been limited by their planar geometries. Here, we use femtosecond laser 3-dimensional (3D) lithography to print a needle onto a nano-SQUID susceptometer to overcome the limits of the planar structure. The nanoneedle coated with a superconducting shell focused the flux from both the field coil and the sample. We performed scanning imaging with such a needle-on-SQUID (NoS) device on superconducting test patterns with topographic feedback. The NoS showed improved spatial resolution in both magnetometry and susceptometry relative to the planarized counterpart. This work serves as a proof-of-principle for integration and inductive coupling between superconducting 3D nanostructures and on-chip Josephson nanodevices.

## Introduction

Superconducting quantum interference devices (SQUIDs) are some of the most sensitive magnetic detectors^1–4^. Nano-SQUIDs with miniaturized SQUID loops or pickup coils can be placed in close proximity to the sample to enhance the magnetic field sensitivity as well as to perform scanning microscopy^5–7^. This is important for studies of samples with small volumes, especially two-dimensional quantum materials and quantum devices fabricated from these materials^8^. While the direct flux sensitivities of nano-SQUIDs are useful in imaging physical quantities such as the magnetization of a ferromagnet, the vortex in a superconductor^9,10^ or the edge current in a quantum spin Hall insulator^11^, susceptometry^12–15^ is indispensable for probing spin correlations^16^ or superfluid densities^17–19^, which are not observable with magnetometry.

Susceptometry performed with nano-SQUIDs utilizes the local magnetic field generated by a small field coil wound around the pickup loop to excite the sample^5^. The response of the sample to the excitation is proportional to its susceptibility and is detected through the pickup loop. Typically, a gradiometric geometry is necessary for the SQUID loop to suppress the mutual inductance between the field coil and the pickup loop^20^. Furthermore, a modulation coil is used to linearize the flux signal with a flux-locked loop^21^. These requirements are only practical when using a multilayered nanofabrication process on a planar substrate.

Although susceptometry is important for investigating 2D spin systems and superconductors, the on-chip nature of a nano-SQUID susceptometer imposes a serious limitation on the spatial resolution. The geometric constraint arising from the planar structure of the nano-SQUID chip prevents close proximity of the pickup and field coils to the sample^22,23^. For this reason, simply making the pickup loop smaller is not effective in achieving a resolution below 500 nm. It is also difficult to use the edge of the chip for height feedback, which is fundamental to the nanoscale spatial resolution in tip-based scanning probe microscopies^24^. A new design is needed to transcend the planar superconducting circuit without compromising the functionality of nano-SQUID gradiometric susceptometers.

In this work, we demonstrate the successful integration of a 3D superconducting nanoneedle with a nano-SQUID susceptometer on a chip. We report the design, fabrication, characterization and test imaging with such a needle-on-SQUID (NoS) probe. The needle was 3D-printed on the nano-SQUID chip with home-built direct-writing photolithography. After sputtering a layer of a superconducting Nb film, we used focused ion beam (FIB) machining to drill a hole at the apex and a slit on the sidewall of the needle. Due to the diamagnetism of the superconducting Nb, the needle collects the flux from a small area underneath the hole while focusing the flux generated by the field coil integrated on the nano-SQUID. Using finite element simulation, we show that the superconducting needle acts as a nanoflux lens to focus the otherwise spreading flux lines both from the field coil and from the sample. The superconducting needle allows us to perform nanoscale topographic and magnetic imaging simultaneously. The spatial resolutions of NoS magnetometry and susceptometry are both superior to those of the nano-SQUID susceptometer without the needle.

### Working principle for the NoS and simulations

**Fig. 1:**
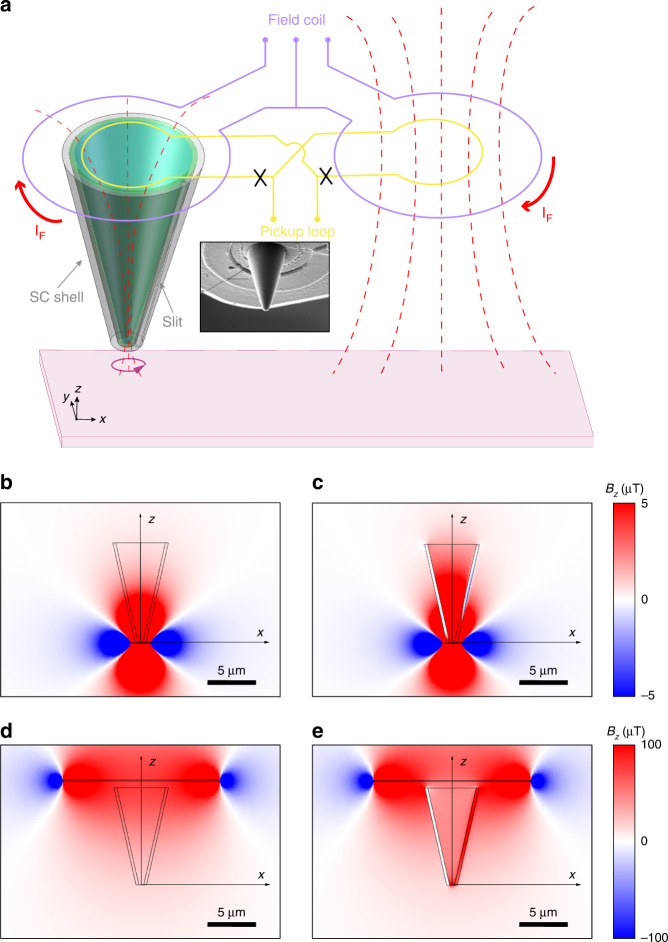
Flux focusing on the nanoscale. **a** Schematic diagram of the needle-on SQUID (NoS) device; the inset is an SEM image of the needle. The needle (green) is a dielectric with no effect on the magnetic field distribution. It is covered with a superconducting (SC) shell (gray) with a hole at its apex and a slit on the sidewall, which breaks the shell into a singly connected topology (see text). The front pickup coil (gray) of a gradiometric SQUID loop is at the base of the needle. It collects the magnetic flux collimated into the needle for reading by the SQUID. The field coil (purple) generates an oscillating magnetic field focused through the needle onto the sample for susceptometry. **b**, **c** Finite element simulation of the magnetic field in the *z* direction (${B}_{z}$) with a 1 μm radius ring with a current of 1 mA placed right beneath the hole (500-nm diameter) of the needle (10-μm height) with non-SC and SC shells, respectively. **d**, **e** Simulated ${B}_{z}$ distribution when applying a current ${I}_{F}$ = 1 mA to the field coil (16-μm diameter) with non-SC and SC shell, respectively. The asymmetry in the field distribution with the SC shell is caused by the slit in the sidewall

The flux-focusing needle we used here has a cone-like structure and a thin superconducting shell with an opening at the apex and a slit on the sidewall (Fig. 1a). The body of the needle is nonmagnetic and is situated directly on top of the front pickup loop of a nano-SQUID susceptometer. The superconducting shell is the main flux-focusing medium because Meissner screening of a superconductor prevents flux lines from going through the shell. The hole at the apex can be drilled to a much smaller size than that of the pickup coil and confines the otherwise spreading magnetic flux from a small area underneath the apex hole. The sharp apex also enables highly sensitive force microscopy. We can perform closed-loop distance feedback so that the apex can be within 10 nm from the sample surface. These qualities of the superconducting needle are fundamentally useful for enhancing the spatial resolution of scanning SQUID magnetometry. However, if there is no slit on the sidewall, the Meissner screening current flows around the hole at the apex, and a small static magnetic field outside the needle is completely expelled. To avoid this, the slit on the sidewall breaks the shell with a hole into a singly connected superconducting sheet^25^. The slit should be made as narrow as possible to minimize flux leakage.

To quantitatively examine the flux focusing effect from the superconducting nanoneedle, we first performed finite element simulations of the magnetic field distribution. We chose a needle height of 10 μm (sufficient for force microscopy) and a base diameter of 5 μm to match the pickup coil of our nano-SQUIDs. The superconducting shell should be thicker than the penetration depth of the superconducting film and was simulated as a medium with a very small relative magnetic permeability (0.01) of 300 nm thickness. Ideally, the slit width should be as small as possible to increase the flux focusing efficiency. However, the redeposition of Nb during the FIB process limited the minimum slit width to ~100 nm. The diameter of the drilled hole determined the spatial resolution and the amount of collected flux signal. We set the slit width to 100 nm and the hole diameter at the apex to 500 nm. The magnetic field distribution from the current ring (1 μm radius) placed under the apex of the needle was expectedly similar to the magnetic dipole field if the needle were nonsuperconducting (Fig. 1b). However, if the shell was superconducting, the *z* component of the magnetic field (${B}_{z}$) would become much stronger inside the pickup loop (Figs. 1c and S1a). This is a strong indication that the nanoneedle collects some of the spreading flux lines generated by the current ring into the pickup loop.

The superconducting needle not only collects flux from the sample but also focuses the flux generated by the field coil. This can be seen from the ${B}_{z}$ generated by the current flowing through the field coil (Fig. 1d, e). The ${B}_{z}$ at the apex of the needle with a superconducting shell is much larger than that with a nonsuperconducting shell (Figs. 1e, S1b). The asymmetry in ${B}_{z}$ is caused by the slit on the sidewall, which allows some flux to leak outside the needle. These results suggest a more concentrated field distribution with the superconducting shell. This property is potentially useful for scanning magnetic resonance imaging^26^ with NoS.

### Nanofabrication of the NoS

**Fig. 2:**
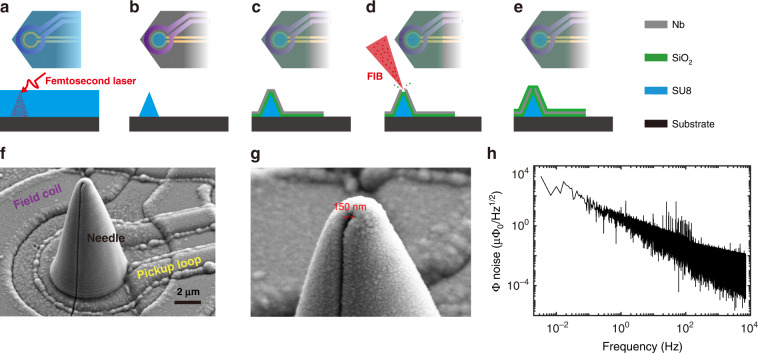
Fabrication and characterization of the NoS device. **a** Printing of 3D needle with a femtosecond laser on a nano-SQUID chip. **b** Removing the unexposed resin with a developer. **c** Deposition of a 200 nm SiO_2_ layer and a 300 nm Nb layer as the superconducting shell. **d** Nanomachining of a hole on the apex and a slit on the sidewall with a focused ion beam (FIB). **e** Deposition of the SiO_2_ protection layer. **f**, **g** Scanning electron microscopy images of an exemplary NoS device. **h** Noise spectrum of an NoS obtained at 4.2 K with a flux-locked loop

We next describe nanofabrication of the NoS (Fig. 2). The main structure of the nanoneedle was 3D printed on the nano-SQUID chip with a home-built direct-writing photolithography system with a femtosecond Ti:Sap laser (Fig. S2). Since it was based on two-photon photopolymerization, femtosecond laser photolithography can produce nanostructures exceeding the diffraction limit of the infrared light employed^27,28^. High resolution is important for achieving a sharp apex on the needle. Tens of microns thick photosensitive resin (SU8) was spin-coated on a nano-SQUID chip (Fig. 2a). The body of a needle with the desired geometry was printed voxel by voxel onto the front pickup loop. The needle was hardened after curing at elevated temperature, which resulting in a Young’s modulus larger than that of SiO_2_. The unwanted resin not exposed to the laser was washed off by the developer (Fig. 2b).

After constructing the main needle structure, we made the superconducting shell on top of it. We first deposited a 200 nm SiO_2_ layer to insulate the SQUID. Then, we deposited a 300 nm Nb layer by magnetron sputtering to serve as the superconducting shell (Fig. 2c). With FIB machining, we sculptured a hole at the apex of the needle and a slit on the sidewall (Fig. 2d). Finally, we deposited another 100 nm of SiO_2_ to protect the shell from possible mechanical damage during scanning microscopy (Fig. 2e). The scanning electron microscopy images showed a fabricated NoS with a sharp apex (Fig. 2f). The hole size of this particular device was 150 nm, and the width of the slit was ~100 nm (Fig. 2g). The fabrication process was reproducible as long as the laser power, exposure time and FIB dose were well controlled. A device fabricated with the same dose looked almost identical (Fig. S4c, d). We have also fabricated NoS devices with larger holes, in which the pattern etched through the Nb was more clearly visible (Fig. S4b). After finishing the device fabrication, we characterized the noise performance of NoS. A successful device exhibits flux noise characteristics similar to those seen before the fabrication process (Fig. 2h). A typical flux noise spectrum for a nano-SQUID chip without a needle is shown in Fig. S5.

### Scanning imaging with NoS

**Fig. 3:**
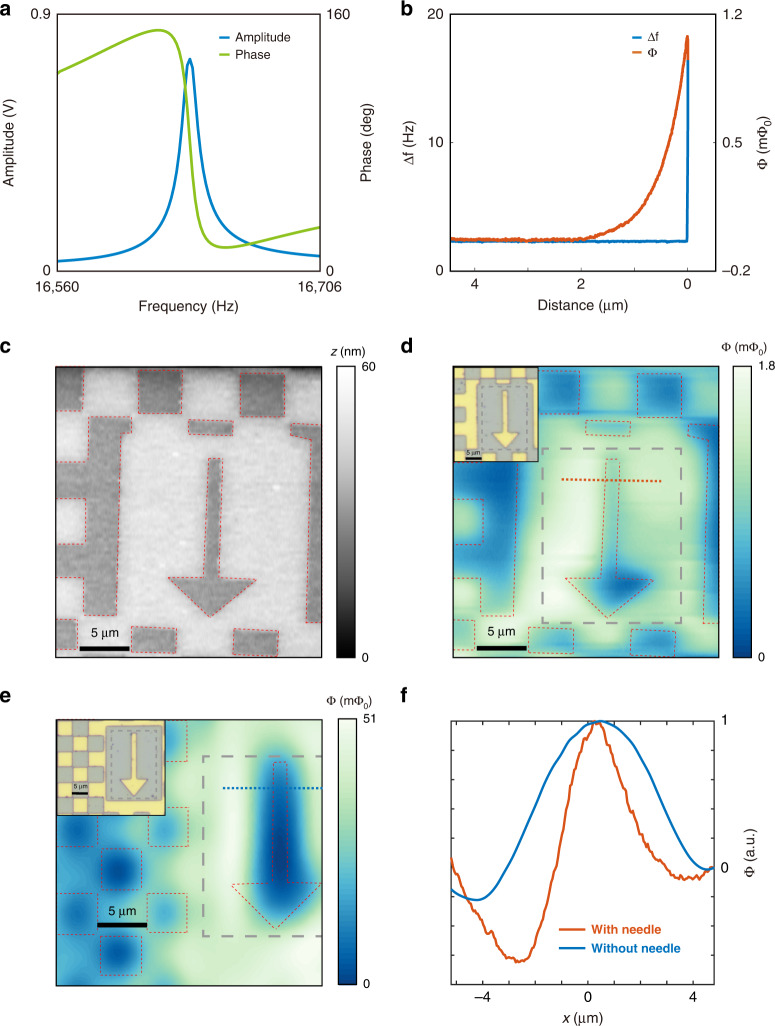
Flux images of a Nb test sample. **a** Amplitude and phase of the tuning fork-NoS assembly as a function of the drive frequency. **b** Approach curve of the resonance frequency shift ($\triangle f$) and magnetic flux ($\Phi$) from the NoS on a Nb test pattern. The NoS has an 800 nm opening on the needle and a 5 μm pickup coil on the nano-SQUID. **c** AFM topography of the test sample measured by the same NoS. The image was obtained with height feedback to maintain a constant distance of several nanometers between the needle and the sample. The light regions are covered with 60 nm of Nb, and the dark regions are film-free. **d** $\Phi$ image obtained simultaneously with **c**. The inset shows an optical image of the scanned region. **e** Magnetic flux image on the same sample at a different area measured by a bare nano-SQUID susceptometer of a 2 μm pickup coil without a needle. The image was obtained at a constant scanning height of 500 nm. The arrow feature is the same as that in **c**. The inset shows an optical image of the scanned region. **f** Linecuts through the arrow patterns (straight dashed lines). $\Phi$ obtained by the NoS was sharper, demonstrating the capability of flux focusing of the needle with a superconducting shell

We demonstrated the imaging capability of the NoS on superconducting test samples. We attached an NoS to a quartz tuning fork (Fig. S3a) for atomic force microscopy (AFM) with the qPlus technique^29,30^. The resonance curve showed that attachment of the NoS shifted the resonance frequency of the tuning fork to ${f}_{0}$ at ~16.6 kHz and reduced the overall quality factor (Fig. 3a). However, it did not affect the phase-locked loop operation of the NoS assembly for the height approach (Fig. 3b) and AFM imaging (Fig. 3c). We obtained the flux signal in a flux-locked loop together with the frequency shift during the sample approach (Fig. 3d). We used the demodulated flux signal at ${f}_{0}$ as the magnetometry signal ($\Phi$). The rise in $\Phi$ started ~2 μm away from the sample and peaked where the needle touched the sample surface.

We set the tuning fork frequency to 1 Hz above ${f}_{0}$ for frequency-modulated AFM after the approach (Fig. 3c). The height difference between the light regions with the Nb film and the dark film-free region was 60 nm, consistent with the film thickness. The simultaneous magnetometry image (Fig. 3d) obtained under an earth magnetic field of 40 μT showed a magnetic contrast consistent with the topography. The region covered with Nb showed a higher signal than the film-free area (defined as zero flux) due to diamagnetic shielding by the film. As a direct comparison, we performed scanning imaging on the same test sample with a nano-SQUID susceptometer chip without a needle (Fig. 3e). The scanned area of the sample was different from that shown in Fig. 3d, but the arrow pattern was the exact same size. Due to the lack of distance feedback without the needle, we fixed the scanning height at 500 nm above the touch-down point to avoid damaging the SQUID and the sample. This distance was several times smaller than the size of the pickup loop, so the resolution was limited by the latter for a planar device^14^. The pickup loop on this nano-SQUID measured 2 μm in diameter instead of the 5 μm diameter used with the NoS. Furthermore, the 10 μm-high needle placed the pickup loop of the NoS much farther away from the sample. A conventional nano-SQUID with a smaller pickup loop and closer pickup loop distance to the sample should exhibit better resolution^5,12^. However, we obtained a much sharper image with the NoS than with the nano-SQUID alone. A line cut through the stem of the arrow showed a sharper variation (Fig. 3f). The result was similar when we compared the arrows of the same pattern (Fig. S6b). These magnetometry images strongly suggest that the superconducting needle on the NoS effectively focused the flux from the sample into the pickup loop.

**Fig. 4:**
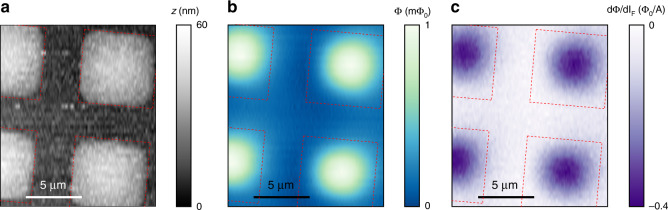
Susceptometry of a Nb square array. **a** AFM topography of a 5 μm Nb square array obtained with a NoS containing a pickup coil with a 5 μm diameter and a field coil with a 16 μm diameter. The opening on the needle is 500 nm. The light squares are covered with a 60 nm layer of Nb. The red dashed lines are the boundaries of the squares. **b** Magnetic flux image of the squares. **c** Susceptometry image obtained simultaneously by applying a 0.5 mA current at 1126 Hz through the field coil. Squares with the Nb film exhibit diamagnetic susceptibility

We now present susceptometry imaging of a superconducting square array with a different NoS. The nano-SQUID and the needle had the same parameters as the first one, except for a 500 nm opening on the needle and a functioning field coil with a 16 μm average diameter. We flowed a 0.5 mA alternating current at 1126 Hz through the field coil and demodulated the $\Phi$ signal at this modulation frequency for susceptibility. The topographic image shows slightly rounded corners on the square and a lower signal-to-noise ratio (Fig. 4a) than the topographic image obtained with the first NoS (Fig. 3a). This may be related to initial scanning outside the sample area, which blunted the apex of the needle. The Nb squares in the magnetometry (Fig. 4b) and the susceptometry (Fig. 4c) images appeared even more circular and smaller than the actual boundaries of the squares. Rounded corners were also seen in a previous study in which a checkerboard square pattern with the same Nb thickness was imaged with a nano-SQUID susceptometer on a chip^14^.

In that work, the susceptometry images were distorted when a pickup loop with a 150 nm diameter was used with a field coil with a 2 μm inner diameter. The large disparities in the sizes of the pickup loops and the field coils may have contributed to susceptometry artifacts when the square array had a size similar to that of the field coil. Since the opening at the needle that collects the flux was more than 10 times smaller than the field coil, we may expect the artifact to be present during NoS susceptometry, yet such an artifact was clearly absent (Fig. 4c), suggesting that the size disparity between flux collection and excitation was avoided. This implied that the detected susceptibility was mainly induced by a localized magnetic field created by the superconducting needle.

We discuss several possibilities for the origin of rounded corners in the magnetic images. First, the characteristic magnetic length scale of the superconducting thin films plays a large part. It is described by the Pearl length $\Lambda =2{\lambda }^{2}/d$^31^$,$ where $\lambda$ is the London penetration depth and *d* = 60 nm is the film thickness. For Nb $\lambda =$ 140 nm at 1.8 K^32^, we obtained $\Lambda =$ 660 nm, which was larger than the opening of the needle. This length scale determined the width of the Meissner screening current, which was ultimately responsible for the magnetic signal. In addition, the Meissner current does not flow against the edge of the square. At the corners, it shifts further away from the edge to reduce its free energy. Therefore, the magnetic images appeared circular rather than squared. Since the susceptibility also comes from (locally) exciting the Meissner current, a large $\Lambda$ can also smear out sharp geometric features even if the superfluid density is uniform across the square.

The geometric effect at the apex of the needle may also have contributed to broadening of the magnetic features. We estimated from the scanning electron microscopy image that the radius of curvature at the apex was ~500 nm after fabrication. The apex may have become blunter after extensive usage (Fig. 4a). This would mechanically shift the opening from the apex, which would allow the in-plane magnetic flux to enter. Such misalignment may affect the magnetic signal when the topography changes quickly, such as at a step edge. Last, since the flux generated by the field coil was focused through the apex of the needle (Fig. 1e), the magnetic field there may exceed the lower critical field of the superconducting shell. This reduces shielding and effectively increases the area of the opening, and thereby decreases the overall resolution in both magnetometry and susceptometry.

In principle, the physical resolution limit of a nanoneedle is determined by the penetration depth of the superconducting shell. Our current work is clearly far from the limit set by the Nb penetration depth. The magnetic behavior of the test sample and the geometric effect of the needle, as discussed above, must be addressed experimentally. On the other hand, we find that FIB drilling with Ga ions has a ‘poisoning effect’ on the Nb superconductivity. As a result, the unshielded area of the needle is always larger than the area milled away by FIB. We leave optimization of the nanoneedle and further enhancement of the spatial resolution for susceptometry to future work. Nevertheless, we argue that this demonstration of the flux focusing capability of a superconducting nanoneedle constitutes a fundamental technical breakthrough for on-chip nano-SQUID susceptometers. Not only are the needle-on-SQUID devices capable of topographic imaging, they also perform susceptometry without resonance artifacts. The flux focusing of the needle allows submicron spatial resolution on nano-SQUIDs with 5-micron-diameter pickup loops, which is significantly better than that of similar nano-SQUIDs without superconducting needles. This proof-of-principle work sets the stage for extensive studies designed to improve the spatial resolution of nano-SQUID susceptometry down to 100 nm and beyond.

## Conclusion

In conclusion, we successfully integrated superconducting needles on nano-SQUID susceptometers via femtosecond laser 3D nanolithography. We demonstrated the flux-focusing capability of the resulting NoS devices. By performing topography, magnetometry and susceptometry imaging of superconducting patterns, we showed that NoS susceptometers are superior to nano-SQUID susceptometers. Our method for constructing 3D superconducting structures on planar superconducting circuits may find broad application in flux-based mesoscopic quantum devices.

## Supplementary information

Supplementary Information

## Data Availability

The data that support the findings of this work are available from the corresponding authors upon reasonable request.

## References

[1] Clarke, J. & Braginski, A. I. *The SQUID Handbook* (Wiley-VCH, Weinheim, 2004).

[2] Maze JR (2008). Nanoscale magnetic sensing with an individual electronic spin in diamond. Nature.

[3] Maletinsky P (2012). A robust scanning diamond sensor for nanoscale imaging with single nitrogen-vacancy centres. Nat. Nanotechnol..

[4] Degen C (2008). Microscopy with single spins. Nat. Nanotechnol..

[5] Kirtley JR (2010). Fundamental studies of superconductors using scanning magnetic imaging. Rep. Prog. Phys..

[6] Granata C (2016). Nano superconducting quantum interference device: a powerful tool for nanoscale investigations. Phys. Rep..

[7] Hilgenkamp H (2003). Ordering and manipulation of the magnetic moments in large-scale superconducting p-loop arrays. Nature.

[8] Aharon-Steinberg A (2021). Long-range nontopological edge currents in charge-neutral graphene. Nature.

[9] Embon L (2017). Imaging of super-fast dynamics and flow instabilities of superconducting vortices. Nat. Commun..

[10] Zhang IP (2019). Imaging anisotropic vortex dynamics in FeSe. Phys. Rev. B.

[11] Nowack KC (2013). Imaging currents in HgTe quantum wells in the quantum spin hall regime. Nat. Mater..

[12] Kirtley JR (2012). Scanning SQUID susceptometry of a paramagnetic superconductor. Phys. Rev. B.

[13] Davis SI (2018). Spatially modulated susceptibility in thin film La_2−x_Ba_x_CuO_4_. Phys. Rev. B.

[14] Pan YP (2021). Improving spatial resolution of scanning SQUID microscopy with an on-chip design. Supercond. Sci. Technol..

[15] Noad H (2016). Variation in superconducting transition temperature due to tetragonal domains in two-dimensionally doped SrTiO_3_. Phys. Rev. B.

[16] Wang SY (2022). Frustrated ferromagnetic transition in AB-stacked honeycomb bilayer. Science Bulletin.

[17] Bert JA (2011). Direct imaging of the coexistence of ferromagnetism and superconductivity at the LaAlO_3_/SrTiO_3_ interface. Nat. Phys..

[18] Bert JA (2012). Gate-tuned superfluid density at the superconducting LaAlO_3_ /SrTiO_3_ interface. Phys. Rev. B.

[19] Wang SY (2022). Oscillating paramagnetic meissner effect and Berezinskii–Kosterlitz–Thouless transition in Bi_2_Sr_2_CaCu_2_O_8+δ_ monolayer. ArXiv.

[20] Huber ME (2008). Gradiometric micro-SQUID susceptometer for scanning measurements of mesoscopic samples. Rev. Sci. Instrum..

[21] Badakhshan M, Mousavi G SM (2018). Flux-lock type of superconducting fault current limiters: a comprehensive review. Phys. C: Supercond. Appl..

[22] Koshnick NC (2008). A terraced scanning super conducting quantum interference device susceptometer with submicron pickup loops. Appl. Phys. Lett..

[23] Kirtley JR (2016). Scanning SQUID susceptometers with sub-micron spatial resolution. Rev. Sci. Instrum..

[24] Finkler A (2012). Scanning superconducting quantum interference device on a tip for magnetic imaging of nanoscale phenomena. Rev. Sci. Instrum..

[25] Little WA, Parks RD (1962). Observation of quantum periodicity in the transition temperature of a superconducting cylinder. Phys. Rev. Lett..

[26] Narasimhan LR, Takigawa M, Ketchen MB (1994). Magnetic resonance of a small platinum particle using an integrated Dc SQUID. Appl. Phys. Lett..

[27] Tanaka T, Sun H-B, Kawata S (2002). Rapid sub-diffraction-limit laser micro/nanoprocessing in a threshold material system. Appl. Phys. Lett..

[28] Kim JM, Muramatsu H (2005). Two-photon photopolymerized tips for adhesion-free scanning-probe microscopy. Nano Lett..

[29] Ng BP, Zhang Y, Wei Kok S, Chai Soh Y (2009). Improve performance of scanning probe microscopy by balancing tuning fork prongs. Ultramicroscopy.

[30] Giessibl FJ (2019). The QPlus snsor, a powerful core for the atomic force microscope. Rev. Sci. Instrum..

[31] Pearl J (1964). Current distribution in superconducting films carrying quantized fluxoids. Appl. Phys. Lett..

[32] Gubin AI, Il’in KS, Vitusevich SA, Siegel M, Klein N (2005). Dependence of magnetic penetration depth on the thickness of superconducting Nb thin films. Phys. Rev. B.

