# Supplementary material for: Flux focusing with a superconducting nanoneedle for scanning SQUID susceptometry
Source: Microsyst Nanoeng. 2023 Jun 12;9:78. doi: 10.1038/s41378-023-00553-9 (PMC10258195; doi:10.1038/s41378-023-00553-9)
Supplement: Supplementary file 1 — Supplementary Information [file 41378_2023_553_MOESM1_ESM.docx]

**Supplementary Information for**

**“Flux focusing with a superconducting nano-needle for scanning SQUID susceptometry”**

This file includes:

1. Supplementary Text of experimental details
2. Figures S1 to S6

# Supplementary Text of experimental details

The noise spectrums (Fig. 2h and Fig. S5) were taken at 4.2 K in a liquid helium dewar. Images by nano-SQUID without needle (Fig. 3e and Fig. S6a) were obtained at 300 mK in a dilution refrigerator and the scanning height was fixed at 500 nm above the touch-down point to avoid damaging the SQUID or sample due to the lack of distance feedback without the needle. The other scanning images by NoS were all taken at 1.8 K with height feedback to maintain a constant distance of several nanometers between the needle and the sample.

# Figures S1 to S6


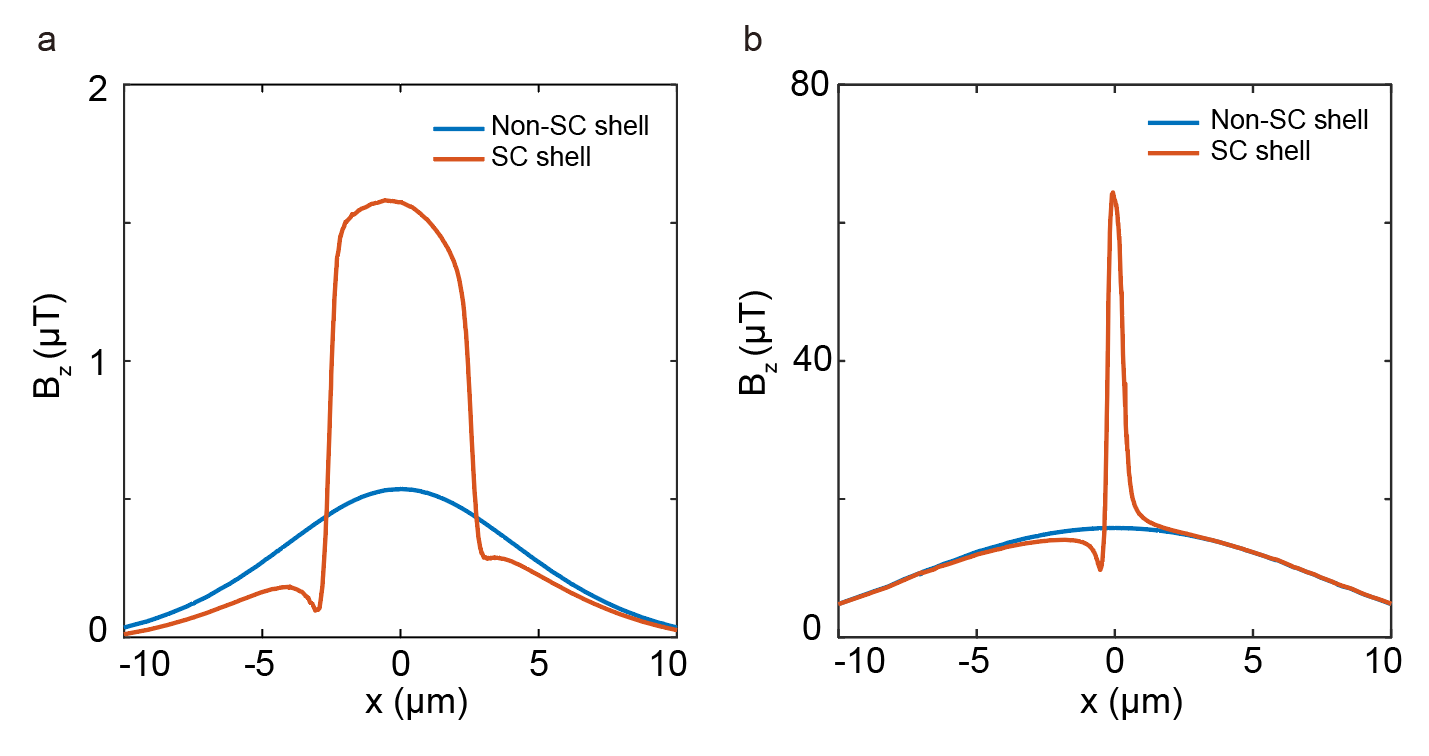


**Figure S1. Finite element simulation of magnetic field distribution.** (a) Distribution of magnetic field in the *z* direction ($B_{z}$) at the plane of the pickup loop (5-μm diameter) when there is a 1-μm-radius ring with current of 1 mA placed right beneath the hole (500-nm diameter) of the needle (10-μm height). The orange (blue) curve is obtained with a (non-)superconducting shell. (b) Simulated $B_{z}$ at the apex plane of the needle when applying a current $I_{F}$ = 1 mA in the field coil. The asymmetry in the field distribution with superconducting needle is caused by the slit on the sidewall.


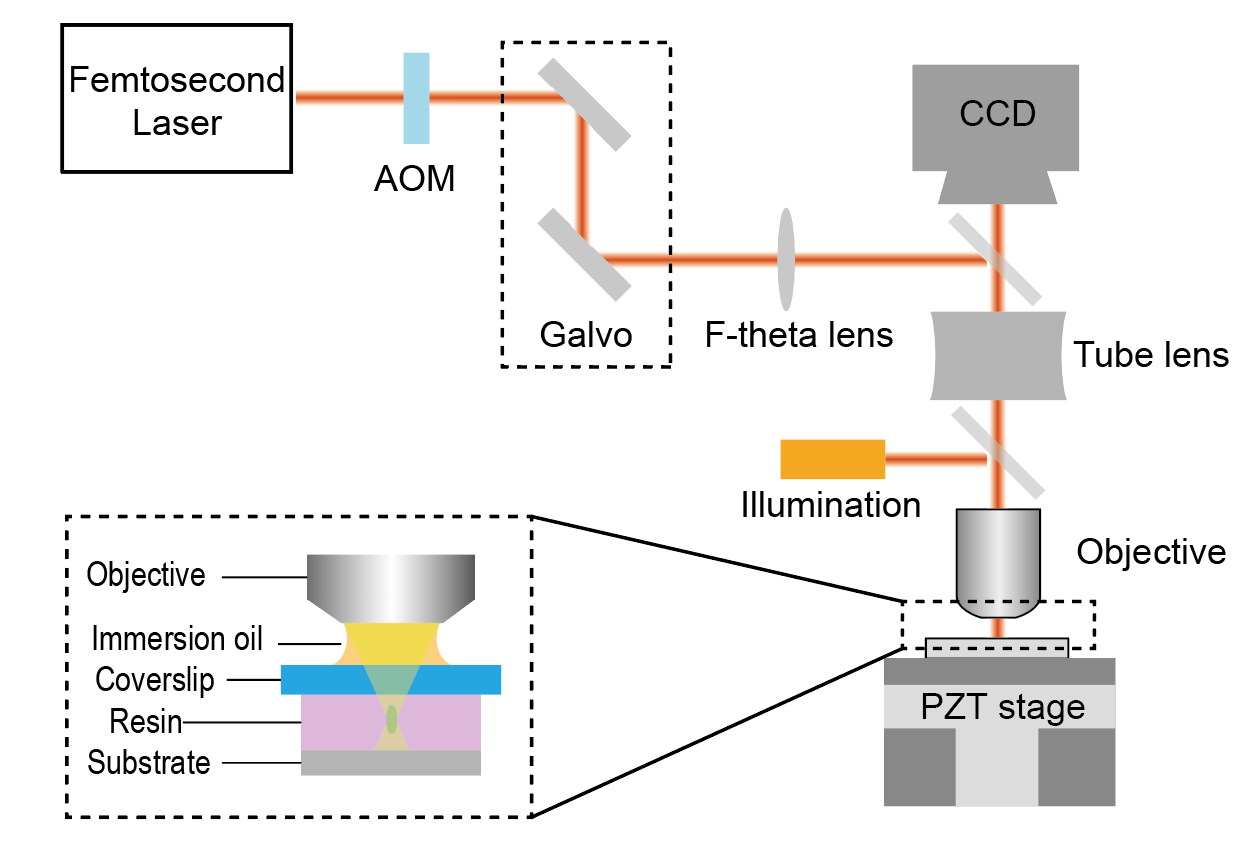


**Figure S2.** **Experimental setup of 3D lithography.** Homebuilt 3D lithography system mainly consists of femtosecond laser (central wavelength 720 nm, pulse width 120 fs), objective (NA=1.25), acoustic optical modulator (AOM) as optical shutter and power attenuator, galvanic mirrors and PZT-controlled translation stage. The laser spot is scanned using galvo mirrors and the sample’s position is moved using a PZT-controlled translation stage. Laser power is adjusted slightly below the damage threshold of the resin to achieve best resolution. The distance of writing lines and layers is set to 100 nm.


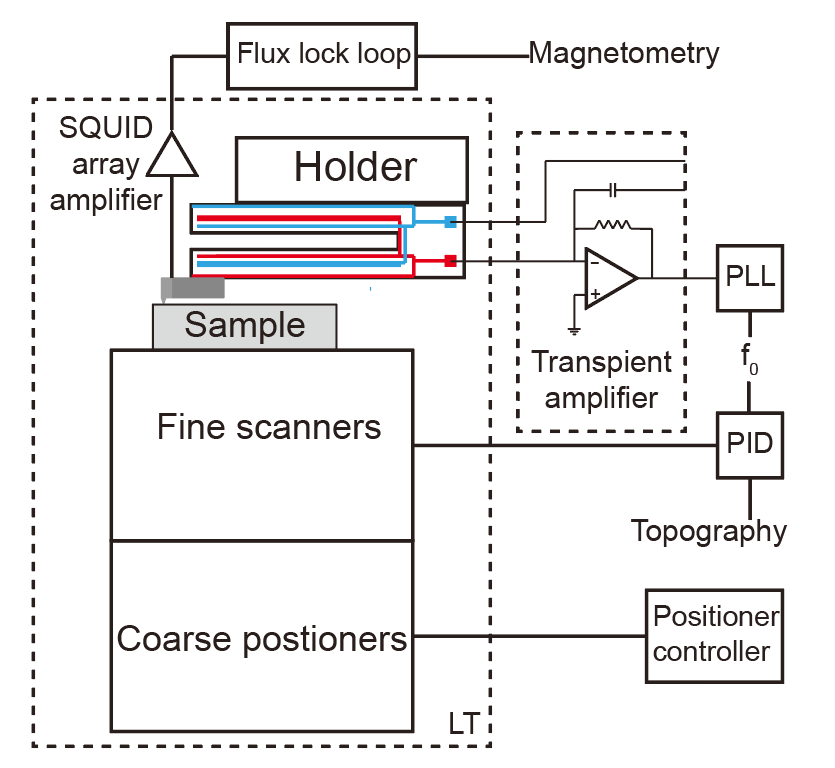


**Figure S3. Diagram of scanning microscopy setup.** Scanning probe setup is composed of atomic force microscope (AFM) for topography imaging and scanning SQUID microscope for magnetic imaging. NoS is attached to a quartz tuning fork for AFM with the qPlus technique. Frequency shift of tuning fork is kept invariant by PID control loop. Flux signal in a flux-lock-loop is demodulated at frequency of tuning fork.


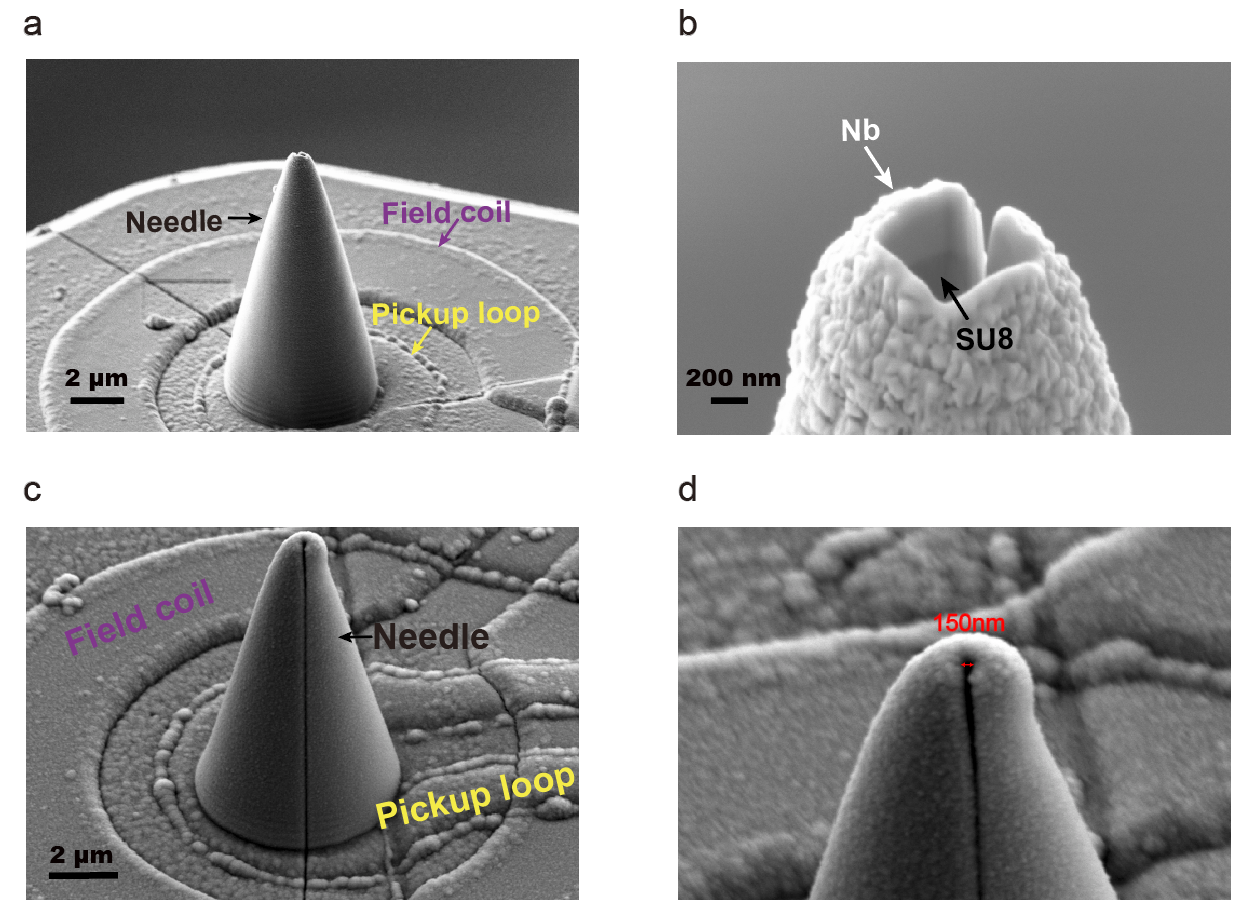


**Figure S4. Scanning electron microscopy images of additional NoS devices.** (a) Image of an NoS device with a 500 nm hole. (b) Zoom-in view of the apex of the needle. The pattern etched through Nb is clearly visible. (c,d) Images of another NoS device with a 150 nm hole.

**
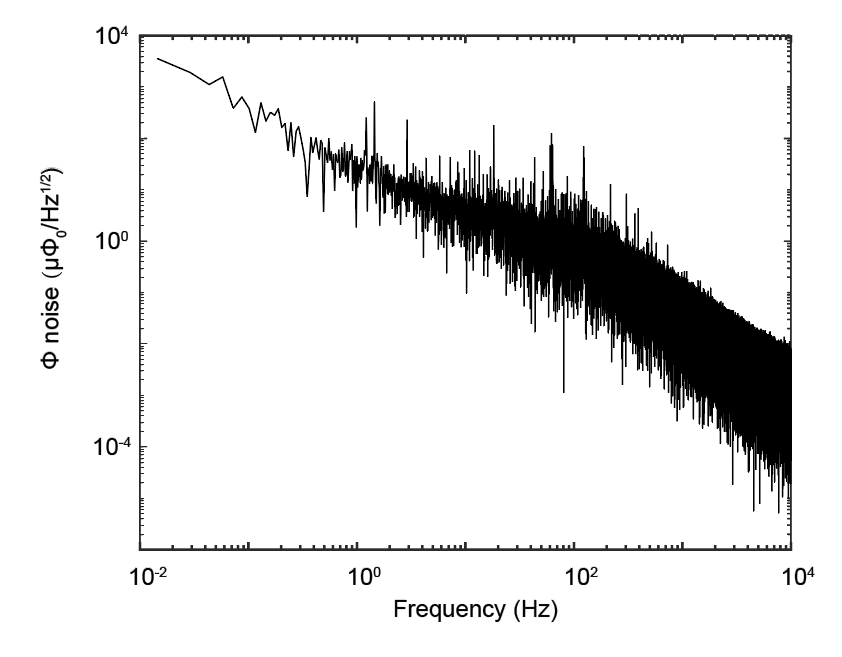
**

**Figure S5. Typical flux noise spectrum of a nano-SQUID chip without needle.** The flux noise characteristics are similar to the NoS devices presented in the main text.


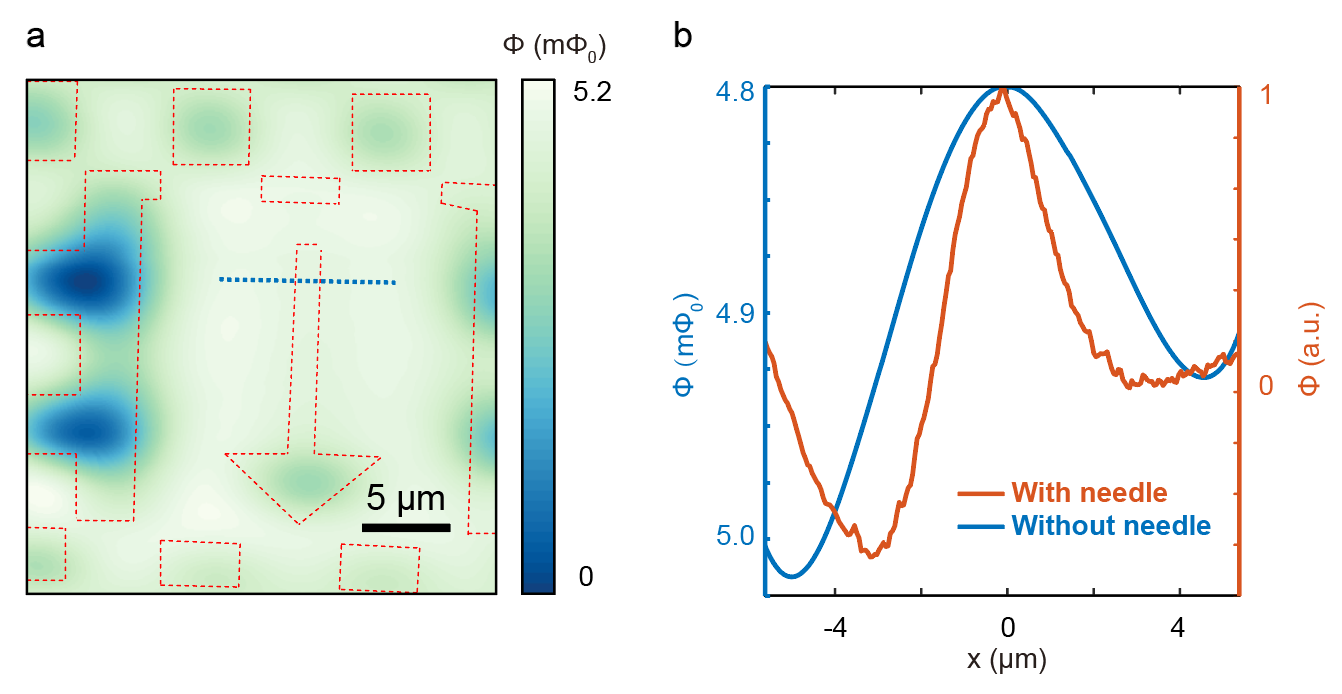


**Figure S6. Flux image of a Nb test sample with a nano-SQUID chip without needle.** (a) Magnetic flux image measured by a bare nano-SQUID susceptometer of 2 μm pickup coil without needle at the same pattern with Fig. 3d. (b) Linecuts through the arrow patterns (straight dashed lines). Φ obtained by NoS is sharper, demonstrating the capability of flux focusing of the needle with a superconducting shell.
